# Giant fibrovascular polyp in the hypopharynx: a case report and review of the literature

**DOI:** 10.1186/s40064-016-3144-y

**Published:** 2016-08-30

**Authors:** Mineko Oka, Rumi Ueha, Takaharu Nito, Tatsuya Yamasoba

**Affiliations:** 1 Department of Otolaryngology Head and Neck Surgery, Tokyo Metropolitan Tama Medical Center, 2-8-29 Musashidai, Fuchu, Tokyo, 183-8524 Japan; 2 Department of Otolaryngology, The University of Tokyo, 7-3-1 Hongo, Bunkyo-ku, Tokyo, 113-8655 Japan

**Keywords:** Fibrovascular polyp, Hypopharynx, Dyspnea, Surgical treatment, Airway management

## Abstract

**Introduction:**

Fibrovascular polyps (FVPs) are benign tumors that commonly occur in the cervical esophagus. Few cases of FVPs of the hypopharynx have been reported, but there has been no English review concerning hypopharyngeal FVPs (hFVPs). Here, we report a case of a vast hFVP, and we also review 13 hFVP cases found in the English literature (PubMed search) including our case.

**Case description:**

A 41-year-old man with respiratory distress and regurgitation of a mass was referred to our hospital. Endoscopic and radiographic evaluations were required for diagnosis. The tumor originated from the hypopharynx and covered almost the entire larynx, which caused the airway to narrow. An emergency surgical removal was performed under general anesthesia with orotracheal intubation, and the tumor was completely removed transorally using a laryngeal endoscope. Pathological examination revealed that the mass was a FVP.

**Discussion and Evaluation:**

We investigated patient characteristics, symptoms, polyp size, treatments, and recurrence of 13 hFVP cases. Regurgitation of a mass, dyspnea, and dysphagia were frequently reported symptoms at presentation. All patients were treated by surgical excision with no recurrence. Airway management is critical and tracheotomies were needed in some cases.

**Conclusions:**

FVPs are often asymptomatic, and they can suddenly cause respiratory distress from laryngeal blockage. Therefore, we emphasize that when such cases are encountered, airway management and surgical treatment should be considered as early as possible.

## Introduction

Fibrovascular polyps (FVPs) are rare benign tumors of the upper digestive tract, and they account for approximately 1 % of all benign tumors in the esophagus and hypopharynx (Avezzano et al. 1990; Sargent and Hood 2006). Most FVPs are located in the cervical very rare (Kim and Shim 2006), and there has been no English review concerning hypopharyngeal FVPs (hFVPs). Most patients are completely asymptomatic, but in some cases, dysphagia, dyspnea, and specifically regurgitation of a mass into the oral cavity may occur. In addition, sudden asphyxia from laryngeal blockage can also occur (Sargent and Hood 2006). Surgical removal is recommended for this disease.

We encountered an extremely rare case of a vast hFVP that caused dyspnea, and we reviewed 13 hFVP cases that have been reported in the English literature, including our case, to verify hFVP characteristics and treatment.

## Case description

**Fig. 1:**
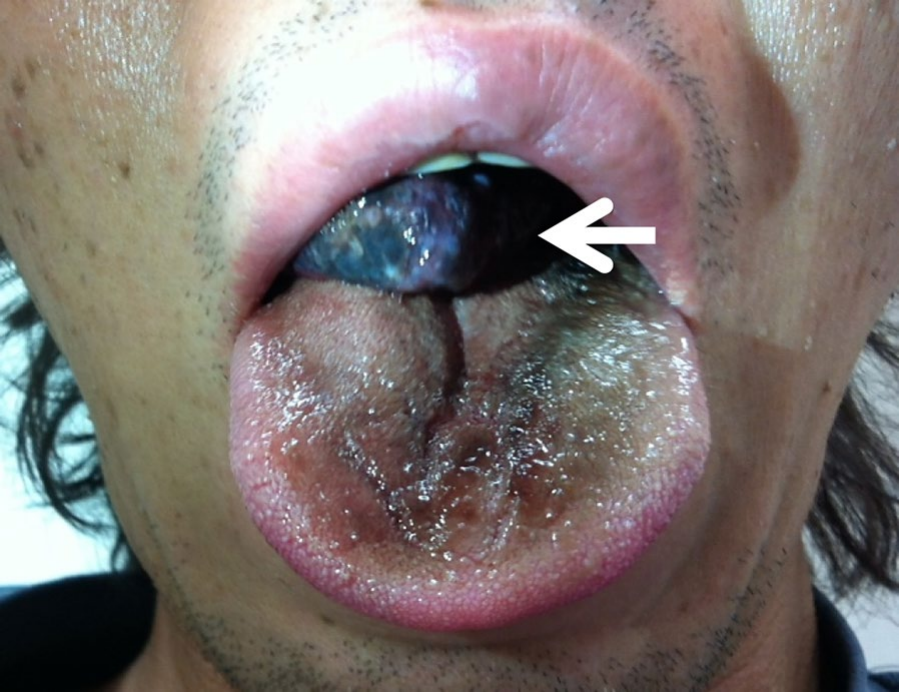
A giant fibrovascular polyp in the mouth (*arrow*)

**Fig. 2:**
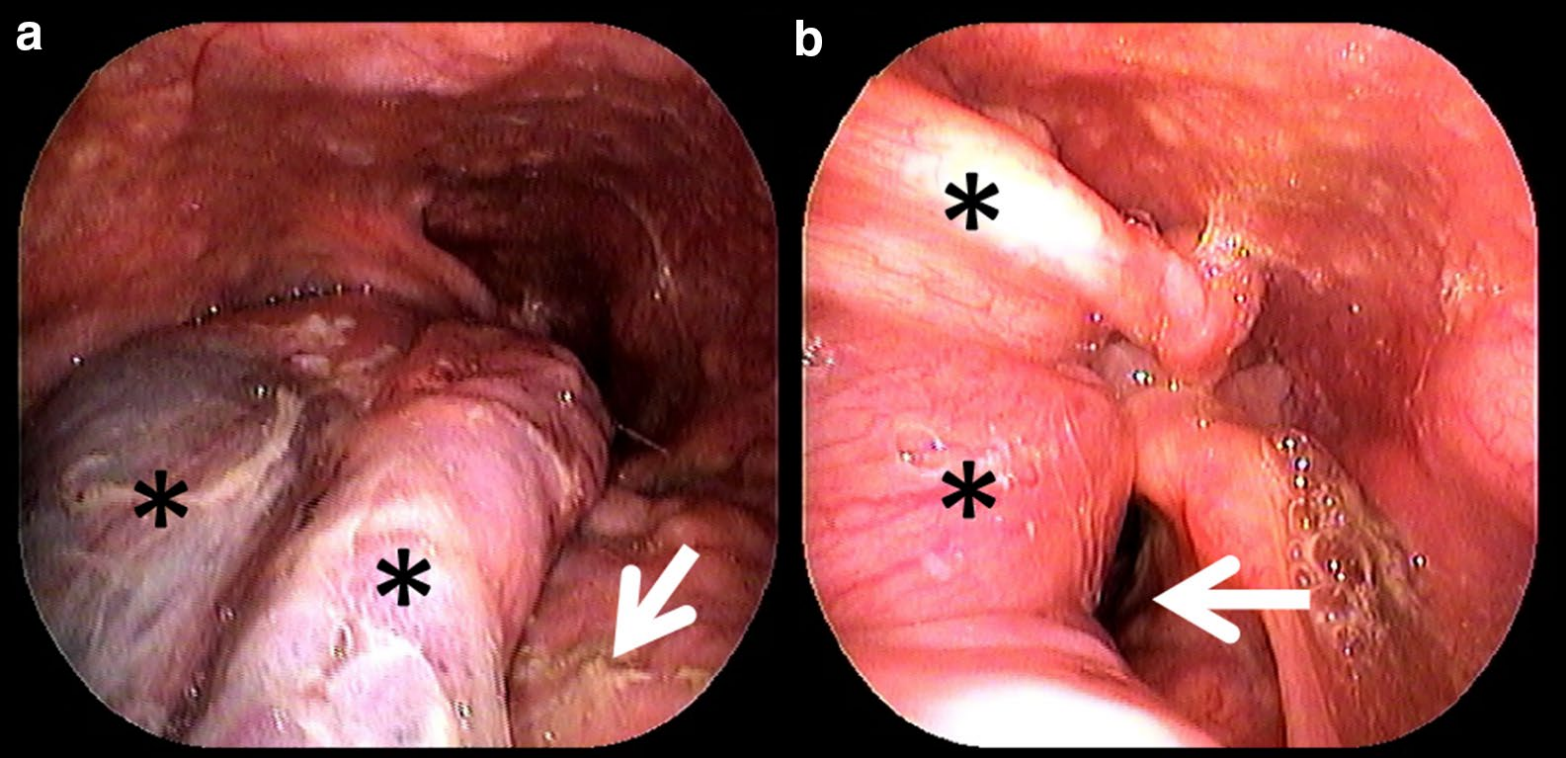
Endoscopic views of the polyp. **a**, **b**: A large polyp (*asterisks*) with two branches from the right hypopharynx on the base of tongue (*arrow* in **a**) covered almost the entire larynx and caused airway narrowing (*arrow* in **b**)

**Fig. 3:**
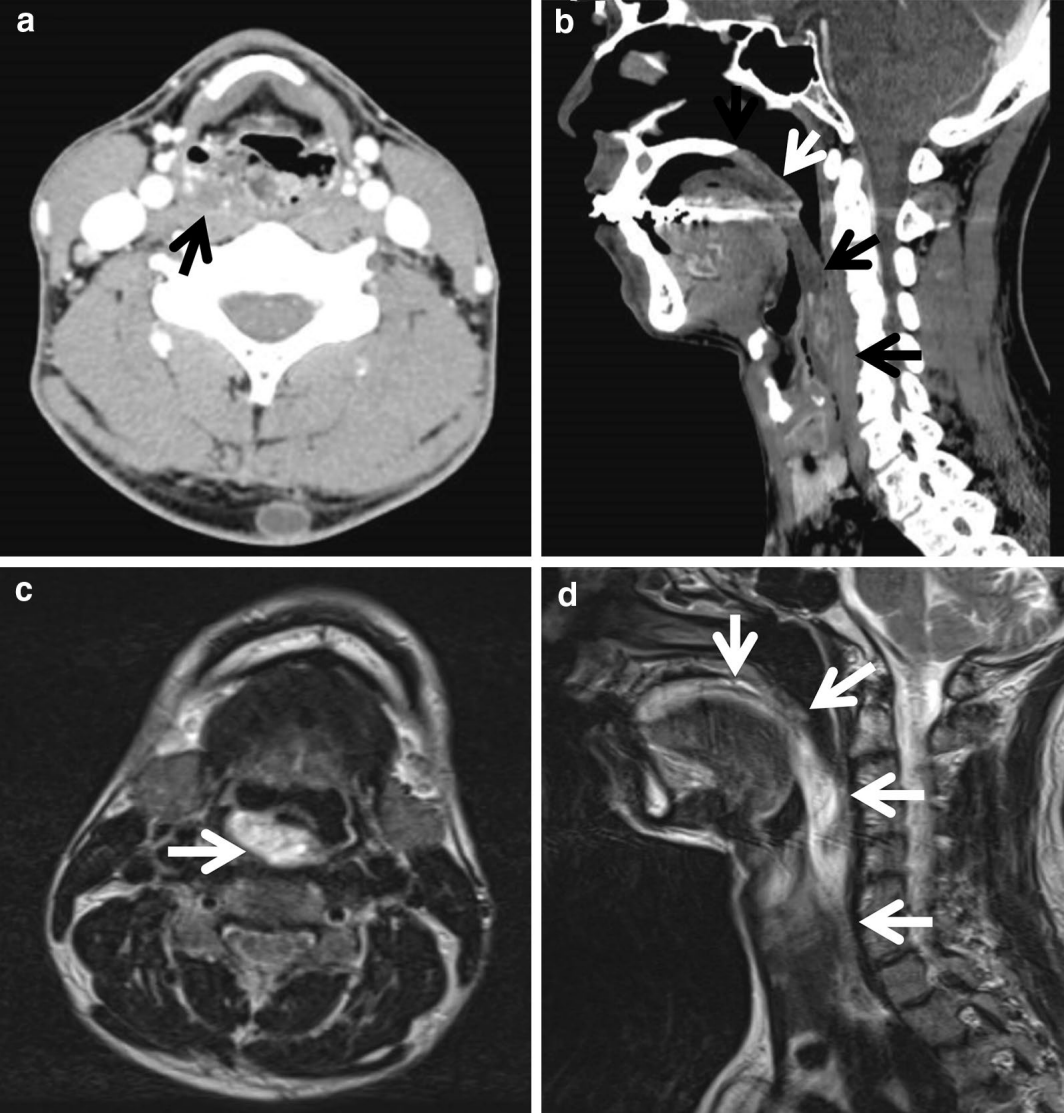
Radiological findings. **a**, **b**: Contrast-enhanced computed tomography scans revealed that the tumor (*arrow*) occupied the oral cavity to the esophagus (**a** axial, **b** sagittal). **c**, **d**: T2-weighted magnetic resonance imaging revealed a high-intensity area within the tumor (*arrow*) (**c** axial, **d** sagittal)

**Fig. 4:**
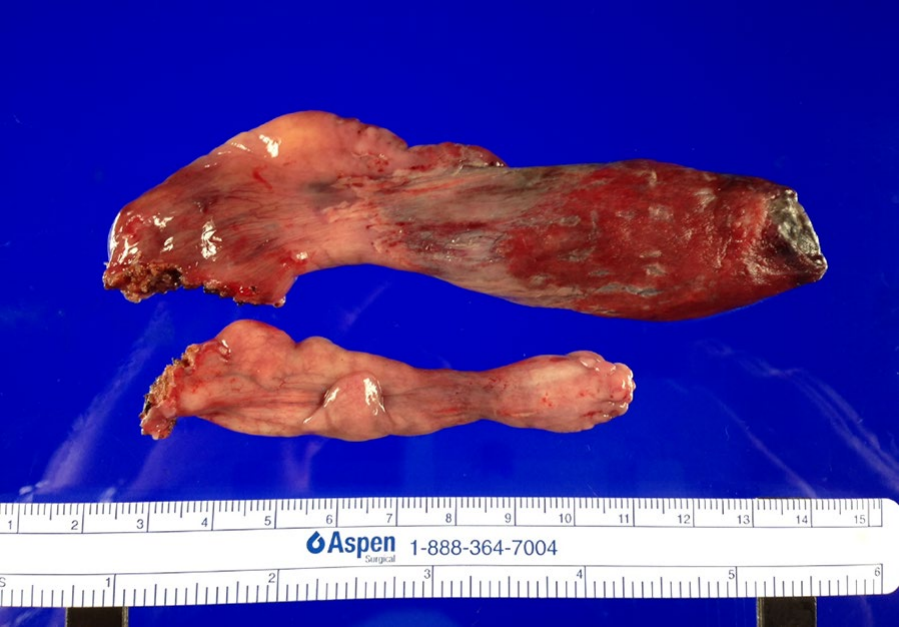
Histological findings. A forked fibrovascular polyp, with branched ends measuring 12 and 8 cm, respectively, was removed

**Fig. 5:**
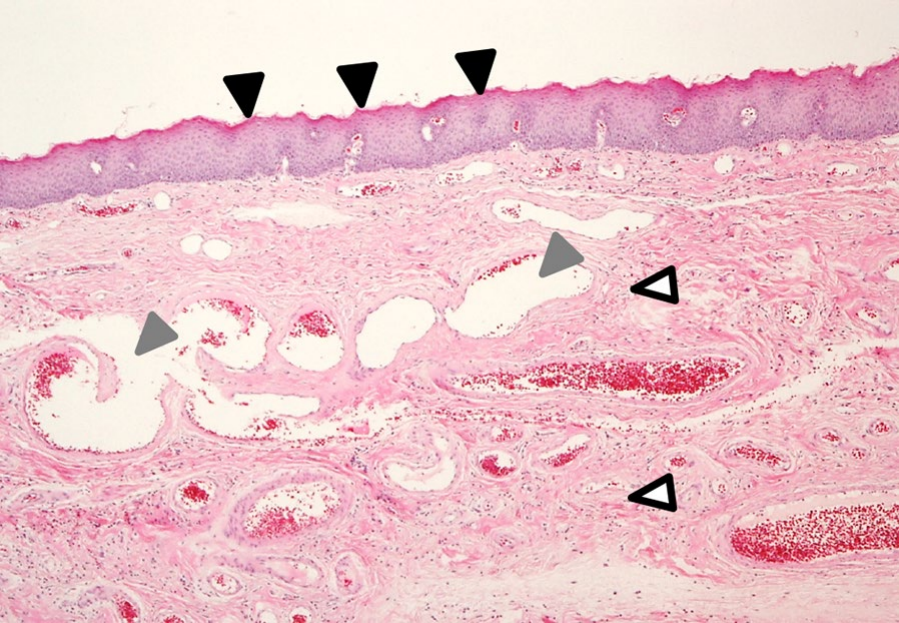
Microscopic findings. The polyp consisted of fibro-adipose tissues (*white arrows*) and blood vessels (*gray arrows*) covered by normal squamous cell epithelium (*black arrows*). Magnification,  × 20; Hematoxylin-eosin stain

A 41-year-old man with no previous medical history was referred to the University of Tokyo Hospital owing to sudden respiratory distress caused by regurgitation of a tumor following vomiting after alcohol intake. A dull, red tumor was protruding from his mouth (Fig. 1), he was experiencing dyspnea in the dorsal position, and his speech was slurred. Endoscopic examination showed a tumor originating in the right hypopharynx and covering almost the entire larynx. The tumor caused almost complete airway obstruction (Fig. 2). Computed tomography (CT) scans showed that the tumor occupied the oral cavity to the esophagus. T2-weighted magnetic resonance imaging (MRI) showed that the tumor had a high-intensity area (Fig. 3). To secure a clear airway, we performed an emergency surgical removal. Orotracheal intubation was successfully performed with endoscopic assistance. Using a Weerda laryngoscope, we could distinctly view the hypopharynx. A pedunculated, soft-tissue tumor originated from the posterior wall of the right hypopharynx, and two branched ends extended into the mouth and thoracic esophagus. We performed a transoral excision completely via laryngeal endoscopy. The body of the tumor was smooth and dark red-brown color, and the two, branched ends of the tumor measured 12 and 8 cm, respectively (Fig. 4). Microscopically, the polyp consisted of a fibro-adipose tissue and blood vessels covered with normal squamous cell epithelium (Fig. 5). Histopathological diagnosis of the tumor revealed a typical FVP. After operation, the dyspnea disappeared. His swallowing function was very good by video-fluorography four days after operation, and he restarted oral intake. The patient recovered with no problems and was discharged eight days after surgery. He has no recurrence in 4 years of follow-up.

## Discussion and Evaluation

More than 100 cases of FVP have been previously reported, but there are few cases arising from hypopharynx and there has been no English review of hFVPs. We searched the PubMed database using two keywords, “fibrovascular polyp” and “hypopharynx”. The search resulted in nine publications (Owens et al. 1994; Nuyens et al. 2004; Zevallos et al. 2005; Kim and Shim 2006; Al-Swiahb and Al-Dousary 2008; Ozdemir et al. 2011; Wang et al. 2011; Pallabazzer et al. 2013; Haytoglu et al. 2015) that included 12 cases in English. We included the case we experienced, and 13 cases in total were reviewed. We investigated patient characteristics, symptoms, polyp size, treatments, and recurrence.

Table 1 shows the details of hFVP for 13 cases. Analysis of available data demonstrated that hFVPs occur with a male to female ratio of 9–4. The mean age at diagnosis was 56 years, with a range of 31–80 years. Almost all patients experienced regurgitation of a mass, and dysphagia was the second most frequent symptom, occurring in 54 % of patients. Dyspnea was also detected in one-third of cases. Other symptoms were reported as follows: vomiting (31 %), laryngeal discomfort (23 %), weight loss (15 %), syncope (8 %), anemia (8 %), and sore throat (8 %). Surgical excision was performed in all cases, and transoral approaches were used in the majority of these cases. Tracheotomy was required in 3 cases (23 %), due to laryngeal obstruction by the regurgitated mass. The mean polyp length was 12 ± 6.2 cm. In four cases, the hFVPs were larger than the 12 cm polyp in our case. In 3 of these cases, pharyngotomy or gastrotomy was used to remove the entire tumor. Recurrence has not occurred after resection in these cases.

**Table 1:** Literature review of hFVPs (n = 13)

Sex (male: female)	9: 4	
Age	56 years; (range 31–80)	
Tumor size	12 ± 6.2 cm	
	Number of cases	Frequency (%)
*Symptom*		
Regurgitation of a mass	11	85
Dysphagia	7	54
Dyspnea	5	38
Vomiting	4	31
Laryngeal discomfort	3	23
Weight loss	2	15
Syncope	1	8
Anemia	1	8
Sore throat	1	8
*Surgical approach*		
Trans-oral (endoscopic/laryngoscopic)	7 (1/6)	54
Trans-cervical	5	38
Gastrotomy	1	8
*Tracheotomy*	3	23
*Recurrence*	0	0

FVPs are rare benign tumors of the upper digestive tract that are rarely encountered in clinical practice. Approximately one-third of large pedunculated polyps (>5 cm) originating in the esophagus and hypopharynx are FVPs (Caceres et al. 2006). Whereas esophageal FVPs are commonly diagnosed in the sixth or seventh decade of life, with the exception of an infant with FVP (Paik et al. 2001), the mean age at hFVP diagnosis was 56 years in the present study. These tumors are reportedly more frequent in males (male: female ratio = 3:1) (Paik et al. 2001), and our review of hFVPs confirmed these data (male: female ratio = 9:4). About 85 % of FVPs are located in the cervical esophagus (Kim and Shim 2006). In addition, rare cases arising from the oropharynx, larynx, or stomach have also been reported (Caceres et al. 2006; Lee et al. 2013; Nascimento et al. 2014; Gupta et al. 2011). Their exact etiologies are not well known. FVPs usually originate as small mucosal tumors adjacent to the cricopharyngeal muscle, and they slowly extend into the esophageal lumen due to the constant downward thrust by food ingestion and peristalsis, sometimes growing to reach the gastric cavity (Kim and Shim 2006). FVPs usually occur as solitary lesions. A case of synchronous hFVPs has been reported (Nuyens et al. 2004), but no previous patient has been described with a forked polyp with two-branches in the hypopharynx as seen in the patient from the present case. Malignant transformation and recurrence for these polyps are reportedly rare, despite some reported cases of recurrence (Lee et al. 2009).

FVPs may remain asymptomatic for many years because of their slow growth. Common symptoms of large FVPs include dysphagia, respiratory distress, and regurgitation of a mass in the oral cavity, which is the most specific symptom that carries risk of aspiration into the airways, resulting in asphyxia (Caceres et al. 2006). In our review, almost all patients experienced regurgitation of a mass, and it is difficult to diagnose FVP without this characteristic symptom. Although there have been reports of sudden death from asphyxiation after regurgitation of a tumor and up to 30 % of patients may die without a correct diagnosis (Timmons et al. 1991), the patient in this case fortunately lived. Various symptoms like laryngeal discomfort, weight loss, and anemia, are observed in patients with FVP.

FVPs can be visualized by endoscopic examination. CT scans and MR imaging can be useful in confirming diagnostic suspicions and in deciding the proper surgical approach. Histologically, polyps are covered by normal squamous epithelium, consisting of adipose and connective tissue, and a well-developed vascular network. The differential diagnosis of FVPs includes leiomyoma, neurofibroma, hemangioma, fibrolipoma, fibromyxoma, hamartoma, fibroma, lipoma, and schwannoma as well as several rare neoplasms, such as carcinoid tumor and chemodectoma (Timmons et al. 1991; Caceres et al. 2006).

Tumor excision is the only treatment for FVP. Sudden asphyxia may occur due to this tumor, and thus, surgical treatment is required as soon as possible after encountering this tumor. Other reasons for excision include the possibility of malignancy and symptomatic improvement. Use of transoral approaches (endoscopic or laryngoscopic) and surgical approaches (transcervical or transthoracic) depend on the FVP location, pedicle vascularity, and size. Identification of the polyp’s pedicle is very important for successful removal of the tumor. Small polyps that are less than 2 cm in diameter with thin pedicles may be removed by the transoral approach. Polyps larger than 8 cm in length or those with thick, richly vascularized pedicles should be removed by surgical excision through a cervical or thoracic approach (Kim and Shim 2006; Wang et al. 2011). However, as shown in the present report, we could successfully remove the tumor by the transoral approach using a laryngeal endoscope, even though the hFVP was 12 cm in length.

Airway management is critical. Tracheotomy or endotracheal intubation allows for controlled ventilation until the polyp can be completely excised. In our review, tracheotomies were performed in 3 patients to protect the airway (Owens et al. 1994; Nuyens et al. 2004; Ozdemir et al. 2011). These three cases had the following features: multiple synchronous FVPs, a long body, and a wide and thick pedicle that might cause laryngeal blockage. In the present case, in spite of double pedicle polyps and a relatively long body, the narrow airway due to the tumor could be secured by orotracheal intubation, and thus tracheotomy could be avoided. When patients experience respiratory distress with regurgitation of the tumor after vomiting, we have to consider FVP. When there is high risk of aspiration or surgical resection cannot be performed rapidly, the airway should be secured by preoperative intubation or tracheotomy.

## Conclusions

We encountered an extremely rare case of a vast hFVP that caused dyspnea, and we also reviewed 13 cases of hFVP. Giant hFVPs are life-threatening because of potential complete airway obstruction and sudden asphyxia due to laryngeal blockage. Thus, when such cases are encountered, airway management and surgical treatment should be considered as early as possible.

## Abbreviations

FVP: fibrovascular polyp
hFVP: hypopharyngeal fibrovascular polyp
CT: computed tomography
MRI: magnetic resonance imaging
